# Kirigami-inspired, highly stretchable micro-supercapacitor patches fabricated by laser conversion and cutting

**DOI:** 10.1038/s41378-018-0036-z

**Published:** 2018-12-03

**Authors:** Renxiao Xu, Anton Zverev, Aaron Hung, Caiwei Shen, Lauren Irie, Geoffrey Ding, Michael Whitmeyer, Liangjie Ren, Brandon Griffin, Jack Melcher, Lily Zheng, Xining Zang, Mohan Sanghadasa, Liwei Lin

**Affiliations:** 1 Department of Mechanical Engineering, University of California, Berkeley, CA 94720 USA; 2 Berkeley Sensor and Actuator Center, Berkeley, CA 94704 USA; 3 Aviation and Missile Research, Development, and Engineering Center, US Army, Redstone Arsenal, Huntsville, AL 35898 USA

## Abstract

The recent developments in material sciences and rational structural designs have advanced the field of compliant and deformable electronics systems. However, many of these systems are limited in either overall stretchability or areal coverage of functional components. Here, we design a construct inspired by Kirigami for highly deformable micro-supercapacitor patches with high areal coverages of electrode and electrolyte materials. These patches can be fabricated in simple and efficient steps by laser-assisted graphitic conversion and cutting. Because the Kirigami cuts significantly increase structural compliance, segments in the patches can buckle, rotate, bend and twist to accommodate large overall deformations with only a small strain (<3%) in active electrode areas. Electrochemical testing results have proved that electrical and electrochemical performances are preserved under large deformation, with less than 2% change in capacitance when the patch is elongated to 382.5% of its initial length. The high design flexibility can enable various types of electrical connections among an array of supercapacitors residing in one patch, by using different Kirigami designs.

## Supercapacitors: Large-area flexibility

Kirigami-inspired “cuts” enable the fabrication of flexible micro-supercapacitors with large-area coverage of the functional component. Flexible electronic devices typically rely on non-flexible—or even brittle—hard functional materials; flexibility is achieved by limiting the areal coverage of the functional component, but this can harm performance. Kirigami—introducing cuts to enable a range of motions—is a promising approach for obtaining flexibility in devices as well as areal coverage. A team led by Liwei Lin from University of California, Berkley now apply laser-induced graphitic conversion and cutting to achieve micro-supercapacitors that can be elongated to over 380% with a functional component areal coverage of over 70%. A range of Kirigami-inspired designs are demonstrated, suggesting the concept might be applied to other flexible functional devices.

## Introduction

**Fig. 1:**
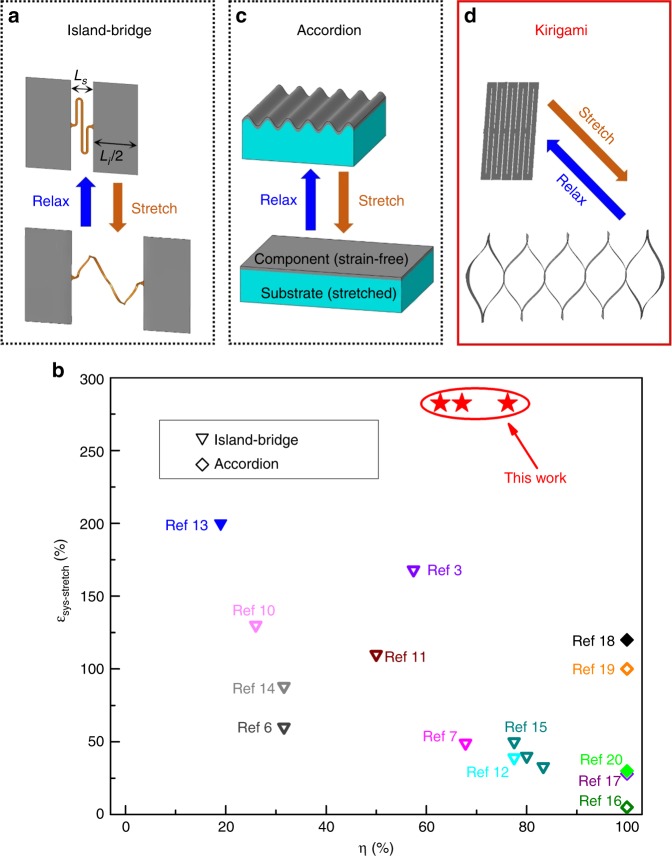
Schematic illustrations of three classes of construct designs employed in stretchable electronics systems. **a** The Island-Bridge construct. **b** System-level stretchability vs. areal coverage of functional components for stretchable systems reported in this paper (highlighted in red) and in other works. Solid markers denote stretchable supercapacitors. Hollow markers denote other electronic systems. **c** The Accordion construct. **d** The Kirigami-inspired construct used in this study

In recent years, stretchable electronics systems have drawn widespread attentions in research and commercialization^1–5^. These systems are mechanically compliant to maintain their electronic performances even when subject to large twisting, bending, and stretching, in applications that require intimate and conformal contacts with curvilinear biological tissues and organs. Reported applications range from epidermal health monitors^6,7^ to implantable optoelectronics platforms^4^, and from wearable energy harvesters^8,9^ to artificial skin-like electronics^10^. Although the aforementioned systems are soft and deformable in the system-level, the functional electronic components in many of these systems are made of hard (e.g., conductive metals) or even brittle materials (e.g., silicon or III–IV semiconductors and their oxides)^1^. As such, the overall desirable mechanics are results of rationally designed constructs, which can provide considerable structural deformability without adding high stresses on the functional parts. One commonly used construct is the “Island-Bridge” model (Fig. 1a), where rigid, functional electronic components (i.e., the “islands”) with side length *L*_*i*_ are separated by spacing *L*_*s*_ among them, and joined by compliant, deformable interconnects (i.e., the “bridges”) to sustain uniaxial stretching deformation by out-of-plane or in-plane unraveling^3,6,7,10–15^. In this system, the areal coverage of functional components *η* can be calculated as *η*=*L*_*i*_/(*L*_*i*_+*L*_*s*_), and the system-level stretchability *ε*_sys_ (defined as the maximum elongation before electronic or mechanical failure) is related to the stretchability of interconnects *ε*_int_ by

1 $$\varepsilon _{{\mathrm {sys}}} = \varepsilon _{{\mathrm {int}}} \cdot \left( {1 - \eta } \right).$$

Since the stretchability of the system *ε*_sys_ and areal coverage *η* are negatively correlated in the “Island-Bridge” model, stretchable electronic systems with this construct (shown as triangles in Fig. 1b) can only achieve high stretchability at the cost of relatively low areal coverage, or vice versa, high areal coverage but limited stretchability. Some other stretchable systems adopt the “Accordion” construct (Fig. 1c)^16–20^. In these systems, thin-film electronics are initially wrinkled due to the release of pre-strain in elastomeric substrates. As the system gets stretched within a limit, the wrinkled films can be flattened out without much axial deformation, therefore providing system-level stretchability. With good layouts, this construct (denoted by diamonds in Fig. 1b) can ideally boast 100% areal coverage, yet the stretchability is limited by the (often quite small) failure strain of the electronics material to be below ~100% of the original length. For stretchable micro-supercapacitors, previously demonstrated works usually adopt one of the two constructs, and consequently show either insufficient stretchability^18,20^ or relatively low areal coverage^13^. Recently, a new class of construct, inspired by the traditional Japanese paper-cutting craftwork (or more commonly known as “Kirigami”, Fig. 1d), has been proposed. In this construct, an intact patch is separated into several deformable segments by rationally designed cuts. Several Kirigami-inspired electronic systems have been reported, including solar cells with tracking functions^21^, implantable bio probes^22^, stretchable graphene transistors^23^, diffraction gratings for LIDAR/LADAR^24^, and piezoelectric strain sensors^25^, etc. The Kirigami construct has also been adopted in stretchable supercapacitors, with reported stretchabilities ranging from less than fifty percent to over several hundred percent^26–28^. In this work, we present stretchable micro-supercapacitor patches adopting well-designed “Kirigami” constructs. As a result, they enjoy the benefits of both “Island-Bridge” and “Accordion” models: they have high areal coverage (76.2%, 62.8%, and 67.1% for three different designs) of functional components and can sustain more than 282.5% elongation with negligible changes in supercapacitor performances. The high areal coverages and high stretchability both come from the design of Kirigami constructs: the deformable segments in the construct, made of functional components themselves, can rotate, bend, and twist out-of-plane to unravel and accommodate significant system-level uniaxial stretching. Although the total elongation of the system may be large, the strains in each segments are small to ensure almost unaffected electronic performances. Compared with previously reported Kirigami-inspired supercapacitors^26–28^, our stretchable micro-supercapacitor patches can be conveniently fabricated in simple and efficient steps by well-controlled laser graphitic conversion and cutting, with the use of one single commercial CO_2_ laser source. In addition, while other Kirigami-inspired supercapacitors can typically sustain a working voltage below ~1 V^26–28^, our stretchable micro-supercapacitor patches can support 10 V or higher by flexibly engineering the layout of electrodes and Kirigami cuts to enable various electrical connections among the array of capacitors in one patch.

## Results and discussion

### Graphitic conversion and cutting

**Fig. 2:**
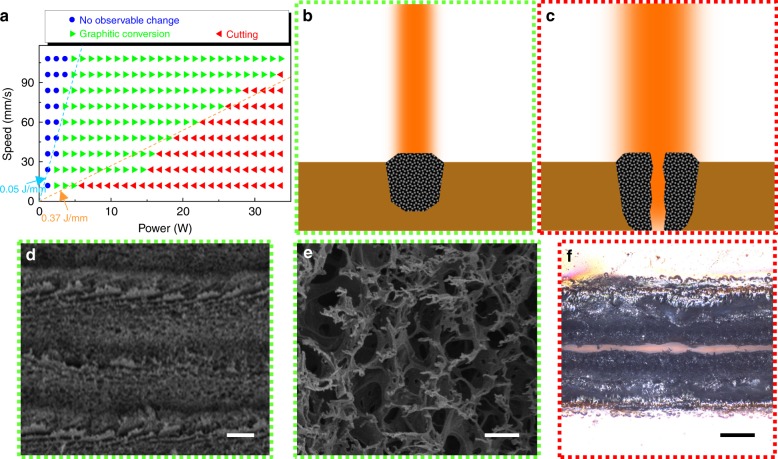
Laser assisted graphitic conversion and cutting. **a** With different combinations of laser power and scanning speed, apparent change (blue dots), graphitic conversion (green dots), or cutting (red dots) can be observed in the polyimide film. The two dash lines denote two threshold linear heat densities. **b** Schematic illustration of graphitic conversion. **c** Schematic illustration of laser cutting. **d**, **e** SEM images featuring microscopic structures resulting from graphitic conversion. **f** Optical image of a cutting slot resulting from laser irradiation. Scale bars: 20 μm in **d**; 2 μm in **e**; 100 μm in **f**

Previous studies have shown that the heat generated from CO_2_ laser can convert commercial polyimide films to conductive graphitic structures (laser-induced graphene, or “LIG”)^29^. Here, we utilize different heating levels from the laser, in order to: (1) set up an appropriate range for the occurrence of graphitic conversion and (2) explore the phenomena when the heating level is high enough to cut through the thin polymer films. For a commercial CO_2_ laser source (*Universal Laser Systems*, wavelength = 10.6 μm), changing the heating level is done through tuning the power of the laser source (unit: W, or J/s) and the scanning speed of the laser head (unit: mm/s). The heating level can be quantified by the amount of heat injected into a unit length on the polyimide film along the scanning direction (often referred to as “linear heat density” or “LHD”, unit: J/mm), which can be calculated from the laser power divided by the scanning speed. Figure 2a summarizes the reactions to a commercial polyimide film (Kapton, Dupont, thickness = 125 μm) when it is irradiated by the CO_2_ laser, with different combinations of power and scanning speed. In this plot, points on the same straight line (with 0 intercept) correspond to combinations that produce equal linear heat densities, while lines of different inverse-slopes represent conditions with different heating levels from the laser source. When the injected heat is insufficient for the photothermal graphitic conversion process to take place (LHD less than roughly 0.05 J/mm, as shown by blue dots in Fig. 2a and in Supplementary Figure S1a), no obvious change on the polyimide film can be observed. Beyond this threshold, the heat generated from the scanning laser converts polyimide near the Kapton film surface to graphitic networks (Fig. 2b and Figs. S1b–h, green dots in Fig. 2a). If the heat output from the laser is even higher than a second threshold (LHD greater than ~0.37 J/mm), the polyimide film is carbonized throughout its thickness so that a continuous cutting slot can be formed (Fig. 2c, red dots in Fig. 2a). The generation of both graphitic patterns and cutting slots are necessary steps in the fabrication of our stretchable micro-supercapacitor patches, as elaborated on in following paragraphs. Scanning electron microscopy (SEM) images in Figs. 2d, e reveal the microscale structures of the highly porous, interconnected graphitic patterns for porous graphitic networks (green dots in Fig. 2a), and an optical image of a laser-cut film is shown in Fig. 2f (red dots in Fig. 2a).

While experimenting different combinations of laser power and scanning speed exhaustively helps define the boundaries among the three “phases” (i.e., no observable change, graphitic conversion, and cutting), we need to obtain the optimal conditions for graphitic conversion and cutting before using them for device fabrication. In following experiments, we tune the LHD by changing laser power, while fixing the scanning speed as 60 mm/s, a reasonable value chosen for easy operation. First, we optimize the conditions of graphitic conversion towards obtaining high-quality electrode materials for supercapacitors: graphitic patterns with abundant microscale pores, low electrical resistance, and good mechanical stability. Optical and three-dimensional (3D) reconstruction images in Fig. S1b–h show the traces made of graphitic networks resulting from ONE laser scan along a straight line at different powers. With laser power increased from 3.6 to 9.6 W (LHD from 0.06 to 0.16 J/mm), larger volumes in the film are affected and converted by the heat, so that the resulting porous graphitic trace gets wider and thicker (Fig. S1b–e). The trace generated at 9.6 W features desirable width and height, and a relatively smooth surface on mesoscale. When the power of the laser is too high (e.g., ~14.4 W or higher), the resulting trace consists of many laminar pieces sticking outwards, likely due to sudden overheating from the intense laser irradiation (Fig. S1f–i). Because these laminae can be peeled off easily, the overall mechanical stability of the graphitic pattern is undesirable. These qualitative observations from optical and 3D reconstruction images help us effectively narrow down the window of optimal graphitic conversion conditions. For a more detailed and quantitative study, we produce many graphitic resistor samples with the same pattern design (6 mm by 60 mm, rectangular strip) at laser powers ranging from 3.6 to 13.2 W, and measure the sheet resistance of each sample using a four-point probe. The top surface and cross-section of these samples are also characterized by SEM, as shown in Fig. S2. When the laser power is less than optimal (e.g., at 4.8 or 7.2 W), the sheet resistance of the generated pattern is relatively high. This is because the heat from laser is not sufficient to reach and convert enough depth of the polyimide film material (Fig. S2b, c). At the optimal laser power of 9.6 W (LHD = 0.16 J/mm), the sheet resistance reaches a minimum at 9.1 Ω/square. Cross-sectional SEM (Fig. S2d) shows that the boundary between the converted graphitic region (thickness ~100 μm) and remaining polyimide is desirably smooth and uniform. It is noteworthy that sheet resistance does not further decrease at conditions with higher laser powers, despite that more materials in the polyimide film become converted (Fig. S2a). This is likely because the excessive heat from laser can both convert deeper regions in the polyimide film (therefore, decrease resistance) and burn out carbonized graphitic structures (therefore, increase resistance) at the same time. As a result, the overall resistance remains almost constant. However, the samples obtained at higher laser powers (e.g., 12 and 13.2 W) have poor mechanical stability. SEM images show that either the jagged boundary between converted graphitic region and remaining polyimide is vulnerable to fracture (Fig. S2e), or that the graphitic networks are already cracked and peeled off with only tiny perturbation (Fig. S2f). Based on the above analyses, the combination of power ~9.6 W and speed ~60 mm/s (LHD = 0.16 J/mm) is used as the optimized condition for producing electrode materials. Our goal for optimizing cutting is to find the condition that guarantees the formation of continuous cut slots while affecting the smallest possible neighboring regions (Supplementary Fig. S3a). When the scanning speed is fixed at 60 mm/s, continuous cutting slots only starts to form when laser power reaches 22.8 W (LHD = 0.38 J/mm). The widths of the cutting slot and the total carbonized regions are 25.2 and 362.1 μm, respectively (Fig. S3d). Since further increasing the laser power (and consequently, heat output from laser) will undesirably increase the widths of total carbonized regions (Fig. S3d–f), we choose laser power 22.8 W (along with speed 60 mm/s) as the optimized condition for defining the Kirigami cutting slots.

### Device fabrication, performance testing and demonstration

**Fig. 3:**
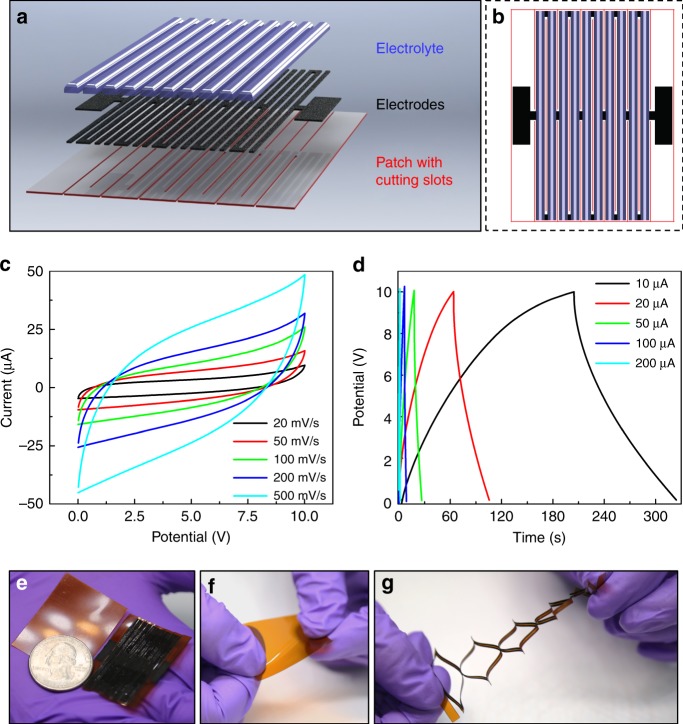
Schematics, performances, and demonstration of a stretchable micro-supercapacitor patch (SMSP) with parallel, alternating offset Kirigami cuts. **a** 3D exploded view and **b** top view illustrations of the layout in the SMSP. Different colors denote three key constituents: Kirigami cutting slots (red), graphitic electrodes (black), and polymer-based electrolyte (semi-transparent blue). **c** Cyclic voltammetry and **d** galvanostatic charge/discharge tests of the SMSP. **e**–**g** Optical image of **f** SMSP and **g** an intact polyimide sheet of the same size in initial and deformed configurations

After the process optimization, graphitic conversion and cutting are adopted as key steps for the fabrication of our stretchable micro-supercapacitor patches (abbreviated as “SMSP” in following paragraphs). First, porous graphitic electrode patterns in the SMSP are generated by irradiating a commercial polyimide sheet (Kapton, Dupont, thickness = 125 μm) with power and scanning speed set as 9.6 W and 60 mm/s, respectively. Then, the laser power is increased to 22.8 W (while maintaining the same scanning speed) to define the Kirigami cutting slots. Some cutting slots constitute the external boundaries of a patch, so that the patch of pre-designed dimensions can be released from a larger Kapton sheet. Other slots (i.e., cutting slots inside the patch) can significantly increases the compliance of the overall patch structure. Finally, a polymer-based electrolyte solution (made from mixing 1 g of polyvinyl alcohol, 1 g of phosphoric acid, and 12 g of deionized water) is applied to selected regions on the patch to make the patch a functional supercapacitor array. Figure 3a, b illustrates the geometric details of a representative SMSP in both exploded 3D view and top view, with the cutting slots (red), the porous graphitic electrodes (black), and the locations to apply electrolyte (semi-transparent blue) highlighted by different colors. The areal coverage of functional components (i.e., combined regions of electrodes and electrolyte) of this SMSP design is 76.2%. The overall size of the patch is 36.5 mm by 28 mm, which is only slightly larger than a US quarter (Fig. 3e). The pre-designed alternating, offset Kirigami cuts transforms the polyimide sheet into five deformable units that are mechanically (and electrically) joined together. Each deformable unit consists of two equivalent parallel plate-like capacitors joined in series. In total, the SMSP has ten equivalent capacitors of the same design in series connection.

We perform standard cyclic voltammetry (CV) and galvanostatic charge/discharge (GCD) tests on our SMSP with an electrochemical workstation (Gamry Reference 600), when the SMSP is in its initial, undeformed configuration (Fig. 3c, d). When the scan rate is set as 100 mV/s, the SMSP yields areal specific capacitance 2.273 mF/cm^2^. As expected, this value is comparable to that reported in ref.^29^, where similar laser-induced graphitic networks were also used as electrodes^29^. The energy density and power density are calculated as 0.32 and 11.4 μW/cm^2^, respectively. At other scan rates ranging from 20 to 500 mV/s, areal-specific capacitances from 1.125 mF/cm^2^ (at 500 mV/s) to 3.654 mF/cm^2^ (at 20 mV/s) are obtained. The capacitance performance of the device can also be confirmed by the various GCD curves in Fig. 3d, corresponding to different charge/discharge current settings.

In order to visually demonstrate the very high deformability of our SMSP, we stretch and twist the SMSP (Fig. 3g) and an intact polyimide patch (with no cuts) of the same size (Fig. 3f). As we expect, the SMSP can be stretched to three times of its initial length and twisted by ~90° easily, whereas the intact Kapton sheet can only be twisted by less than 30° with negligible increase in length, even when much higher force and torque are applied. This comparison qualitatively confirms our expectation that introducing pre-designed Kirigami cuts can increase the deformability of polyimide patches significantly.

### Analysis of buckling determinism

As shown in Fig. 3g, the elongation of our SMSP is contributed by buckling-initiated out-of-plane deformation. Traditionally, structural buckling is associated with an unpredictable state of chaos, and is, therefore, discouraged and circumvented by most engineers. In many cases across length scales, buckling was treated as the first route to failure, ranging from bridges^30^ and pressure vessels^31^, down to MEMS structures^32^. In recent years, many researchers have started to analyze buckling and to make use of controllable buckling instability, especially in developing exotic mechanical metamaterials, stretchable electronic systems, and unconventional soft sensors and actuators^33^. To utilize structural buckling for achieving stretchability, we need to confirm that the buckling in our SMSP is deterministic and predictable. In our study, we have performed finite element analysis (FEA) using the linear perturbation module of a commercial FEA package (ABAQUS) to calculate possible modes of buckling in our SMSP^3,7,34^. The Young’s modulus of polyimide is set as 2.5 GPa, and effective modulus of the graphitic electrodes as 300 MPa, respectively, in FEA. Supplementary Figure S4a shows the computed buckling shapes corresponding to the first four buckling modes in top, side, and 3D views, where the colors denote normalized out-of-plane displacement. The shape of Mode 1 is featured by all the five deformable units rotating in the same direction, and the cut slot in each unit opening slightly. This buckled shape is anti-symmetric about the central axis (denoted by dash-dot lines), as is apparent in the top and side views. In other words, if a point moves downward, its reflected point about the axis of symmetry moves upward by the same amount. Out of the first four modes, Mode III also has anti-symmetric buckling shape, whereas the buckling shapes of Mode II and IV are symmetric about the central axis. The computed critical buckling strains *ε*_crit_ corresponding to the first four modes are shown in Fig. S4b. Because the critical buckling strain for Mode I (*ε*_crit_=0.72%) is significantly lower than those of the other three modes (by 28–58%), the SMSP will buckle following the route of Mode I when it is elongated slightly (by 0.72%), before any other modes could possibly occur. Figure S4c shows the deformation energies of the SMSP as a function of elongation (from 0 to 100%) following the different routes defined by the four modes. Apparently, the deformation energy for Mode I is consistently smaller than that for other modes in ALL shown stages of elongation. Therefore, following the principle of minimum deformation energy, the SMSP should always deform along the route defined by Mode I, and that a snap-through to another mode is not expected to occur^35,36^. Even if the deformation of the patch deviates from Mode I temporarily, it is expected to “snap back” to this mode quickly. As supported by both arguments (i.e., critical buckling strain and deformation energy) and later confirmed by experiments, Mode I is the only dominant mode expected to take place.

### Post-buckling deformation

To systematically analyze the post-buckling deformation (i.e., elongation) of the SMSP samples, a failure criterion for the graphitic electrodes is required. Here, we designed an experiment and performed corresponding finite element analysis in order to set up a simple strain-based failure criterion, since well-recorded failure properties of this unconventional material are not readily available. Supplementary Figure S5a illustrates the experiment setup, where a test sample with laser converted graphitic pattern is connected to a four-probe station. This station can characterize the resistance of the test sample (with the shape of a rectangular beam) clamped at two ends (in dash box). The initial length and resistance of the sample are *L*_0_ and *R*_0_, respectively. When the distance between the two ends is reduced by an amount −Δ*L*, the sample bends upwards or downwards and results in a measured change in resistance Δ*R*. From the relationship between Δ*R*/*R*_0_ and Δ*L*/*L*_0_ plotted in Fig. S5b, we notice that the increase in resistance remains very small (Δ*R*/*R*_0_ < 2%) even when the end-to-end distance of the sample is shrunk by half (Δ*L*/*L*_0_ = −50%). However, further compressing the sample (either bent upwards or downwards) beyond Δ*L*/*L*_0_ = −50% will result in a significant, irreversible increase in resistance, most likely due to the microscale fracture within graphitic networks. Figure S5c shows the deformation (both upward and downward bending) of the sample with end-to-end distance reduced by 50%, as observed in experiment and predicted by FEA with very good agreement. At this level of deformation, the computed maximum absolute principal strain *ε*_max_ (via FEA) in the graphitic layer is 5.5%. Based on this result, we use “maximum absolute principal strain *ε*_max_ in the graphitic material reaching 5.5%” as the failure criterion in all following analyses.

**Fig. 4:**
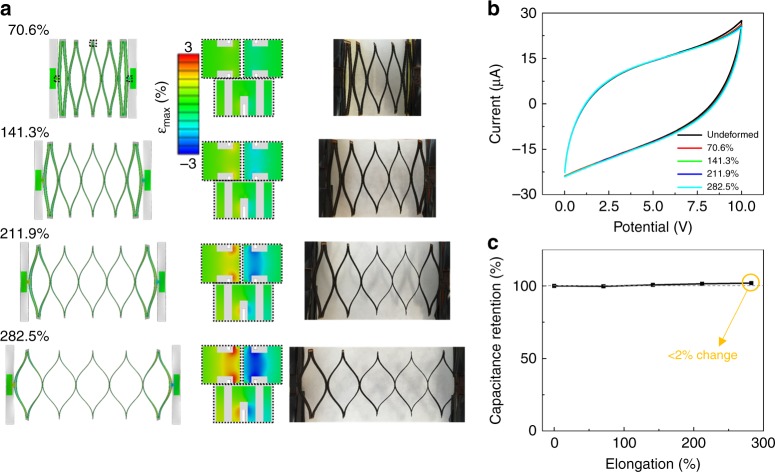
**Post-buckling deformation and influences to electrochemical performances. a** Post-buckling deformation of a representative SMSP, featuring FEA predictions (left column) and optical images (right column) of the SMSP at four deformation stages. The center column highlights strain distributions at selected sites with large deformation at each stage of deformation. Color in FEA images denotes computed maximum absolute principal strain. **b** Cyclic voltammetry curves and **c** capacitance retentions of the SMSP under different elongations. The scan rate is 200 mV/s

Fig. 4a shows the deformation of the SMSP at four elongation stages (70.6%, 141.3%, 211.9%, and 282.5%), as predicted by FEA (left column) and observed in experiments (right column). The modeling results agree very well with the optical images in almost all geometric details. As predicted, SMSP deforms following the route defined by Mode I. When the SMSP is stretched beyond the onset of buckling (i.e., elongated by more than 0.72%), each deformable unit in the patch continues rotating, and the cut slots in each unit opens up further, such that the initially straight segments pop out of plane and bend considerably to accommodate the applied stretch. Both the rotation (and subsequent twisting) and the bending contribute to the overall stretchability of the SMSP. Because of this deformation mechanism, the actual elongation in the functional electrodes is quite small even when the SMSP is stretched by 282.5% to almost four times of its initial length.

In the center column of Fig. 4a, magnified views show the strain mapping at three representative regions that have the most significant strain concentration in the SMSP. Even in these regions, the maximum absolute principal strain (*ε*_max_ < 3.0%) in the graphitic electrodes is always lower than 3.7%, even for 282.5% of elongation. Because the strain in electrode material is quite small, we expect negligible degradation in electronic performance induced by the deformation of SMSP. In experiments, we find indeed that the five CV curves corresponding to different elongation stages overlap very well (Fig. 4b). The change in capacitance retention is very small (less than 2%; Fig. 4c) even with significant overall stretching deformation (0–282.5%). Notably, this<2% change in capacitance is smaller than most reported stretchable supercapacitors, despite that our elongation of device is usually greater. Both the mechanical analyses and the electrochemical testing results above prove that our SMSP with this Kirigami construct can maintain full performances under extreme deformations. While the elongation was limited by the range of our stretching platform at 282.5% in experiments, the FEA model predicts further deformation of SMSP up to 600% elongation, as shown in Supplementary Figure S6. In the region with the most severe strain concentration (highlighted in magnified views), *ε*_max_ exceeds 5.5% when the overall elongation is greater than 510%. Therefore, we predict the stretchability of our SMSP to be ~510%, which is comparable to the value (500%) of the reported most stretchable supercapacitor^27^. Additionally, a rough stretching test by hand (Figure S7) demonstrates that mechanical integrity of the SMSP can be maintained at up to over 730% elongation, while rupture of the structure occurs at 775% elongation.

### SMSPs with other Kirigami-inspired designs

**Fig. 5:**
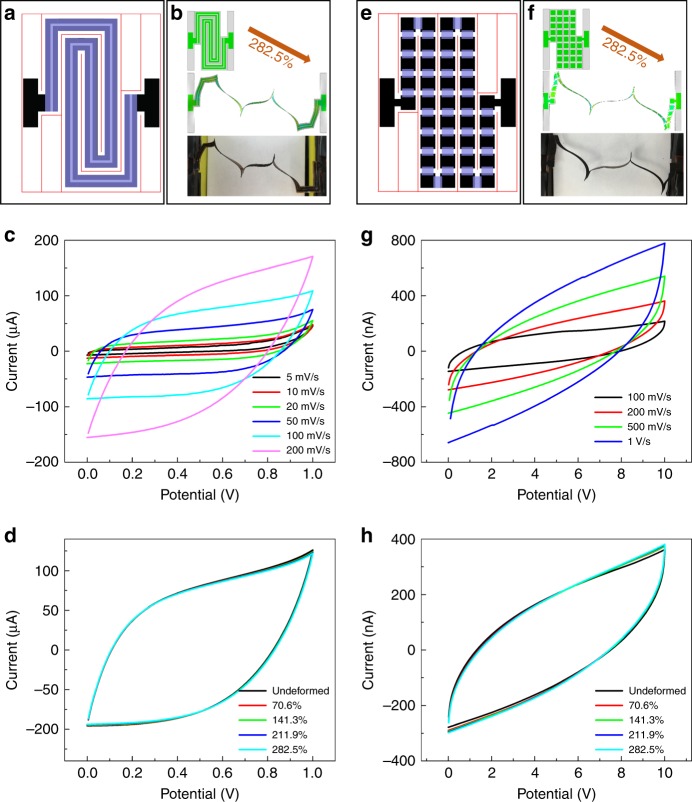
Design, deformation, and performances of SMSPs with two other Kirigami-inspired designs. Namely , **a**–**d** the “double spiral” design and **e**–**h** the “zigzag serpentine” design. In **a** and **e**, colors denote the layout of electrodes (black), Kirigami cuts (red), and electrolyte (semi-transparent blue) in each design. **b** and **f** show the deformations of SMSPs predicted by FEA (top: undeformed; middle: elongated by 282.5%) and observed in experiments (bottom: elongated by 282.5%). **c** and **d** show the cyclic voltammetry curves for the SMSP with “double spiral” design measured at **c** different scan rates and **d** different stages of deformation; **g** and **h** show the cyclic voltammetry curves for the SMSP with “zigzag serpentine” design measured at **g** different scan rates and **h** different stages of deformation

In the aforementioned SMSP with ten equivalent capacitors connected in series, we employed the most well-known Kirigami pattern, the “alternating offset cuts”, to obtain high system-level stretchability. Inspired by the abundant existing Kirigami cut patterns, we have also designed equally stretchable micro-supercapacitor patches with various mechanical constructs and electrical connections. Figure 5 illustrate examples of SMSPs with the same aforementioned overall size of patch (36.5 mm by 28 mm) but a distinct layout of cutting slots, electrodes, and electrolyte. The SMSP in Fig. 5a–d adopts a “double spiral” design. In this patch, the pair of parallel electrodes forms a path similar to two spirals joining together at the center. The areal coverage of functional components is 62.8%. This SMSP with two very long electrodes can be considered as one equivalent capacitor that can sustain 1 V. Figure 5c shows the CV curves of this SMSP in its undeformed configuration. At a scan rate 10 mV/s, the specific capacitance is measured as 0.57 mF/cm^2^. This lower value than that in Fig. 3 is likely due to the much higher ionic resistance from the long electrode segments. When this SMSP is subject to a tensile load, the segments near the center buckle out of plane, and accommodate the stretch by rotating, bending, and twisting in subsequent post-buckling deformations (Supplementary Figure S8). Similar to the SMSP in Fig. 5, this SMSP with double spiral design can maintain its full electrical performance (<0.9% change in capacitance retention) with up to 282.5% elongation (Fig. 5b), as justified by CV tests performed at different stages of deformation (Fig. 5d).

In addition to the two SMSPs described here, the Kirigami concept can also be used in designing stretchable supercapacitor patches that can sustain higher voltage. Figure 5e–h features an SMSP with “zigzag serpentine” design and areal coverage 67.1%. The 37 square-shaped electrodes in this patch constitute a chain of 36 equivalent capacitors connected in series, and arranged in a path resembling a zigzagged serpentine. When the patch is stretched uniaxially, the Kirigami cutting slots in it allow different segments of the “serpentine” to buckle, rotate, bend, and twist (Supplementary Figure S9). The CV results in Fig. 5g yield a specific capacitance 0.531 mF/cm^2^ at a scan rate 200 mV/s, when the scanning range is 0–10 V. We expect that this SMSP can potentially sustain a much higher voltage range (up to ~36 V), yet tests to validate this exceed the limit of our electrochemical workstation. Like the other two SMSPs described in previous paragraphs, the performance of this SMSP is also not affected by large uniaxial stretching. The change in capacitance retention is less than 1.8% throughout different stages of elongation up to 282.5% (Fig. 5h).

## Conclusions

This work presents a series of stretchable micro-supercapacitor patches with constructs inspired by the traditional Japanese craftwork, Kirigami. By adopting this design concept, we have achieved simultaneously high system-level stretchability and high areal coverage of functional components in the patches. These stretchable patches can be fabricated in simple steps by graphitic conversion and laser cutting, using the same commercial laser cutter with different heating level settings. Because the compliant Kirigami-inspired structure can easily sustain large deformation with very small strain in electrodes, the electrical performances of the patches are uncompromised even under extreme elongation. Various electrical connections among supercapacitors in one patch can be obtained by using different Kirigami designs, with different layouts of electrodes and cuts. In addition to micro-supercapacitors, we believe the proposed Kirigami-inspired designs can also provide a new way in the development of many other stretchable electronics and bioelectronics systems, especially for those requiring both high deformability and high functional areas.

## Materials and methods

### Device fabrication and testing

The graphitic electrodes and Kirigami cuts in the micro-supercapacitor patches were generated by irradiating commercial a polyimide sheet (Kapton, Dupont, thickness = 125 μm) with CO_2_ laser (Universal Laser Systems, wavelength = 10.6 μm) at different optimized power and speed settings (9.6 W, 60 mm/s for forming graphitic electrodes, and 22.8 W and 60 mm/s for cutting, respectively). The electrolyte (mixture of 1 g of polyvinyl alcohol + 1 g of phosphoric acid + 12 g of deionized water) is applied manually to selected regions on the patch. The performances of supercapacitors were measured on an electrochemical workstation (Gamry Reference 600).

### Finite element analyses

3D FEA simulations were performed using a commercial simulation software (ABAQUS) to analyze the buckling behaviors and the post-buckling deformation of the SMSPs. The SMSPs composed of unconverted Kapton (polyimide, Young’s Modulus *E* = 2.5 GPa, Poisson’s ratio *ν* = 0.34) and graphitic electrodes (*E* = 300 MPa, *ν* = 0.3, estimated values) were modeled with quadrilateral shell elements (S4R)^3,7^. The overall deformations of the graphite/Kapton bilayer are mostly determined by the mechanical properties of polyimide. For different regions in the SMSP, the “Composite Layup” function was used to correctly define the thicknesses and properties of materials of each constituting layers. Since the solution-based electrolyte has almost no influence to the mechanical properties of the SMSP, it was neglected in the FEA modeling.

## Electronic supplementary material

Supplemental Material
