# Supplementary material for: Kirigami-inspired, highly stretchable micro-supercapacitor patches fabricated by laser conversion and cutting
Source: Microsyst Nanoeng. 2018 Dec 3;4:36. doi: 10.1038/s41378-018-0036-z (PMC6275159; doi:10.1038/s41378-018-0036-z)
Supplement: Supplementary file 1 — Supplemental Material [file 41378_2018_36_MOESM1_ESM.docx]

**Kirigami-Inspired, Highly Stretchable Micro-Supercapacitor Patches Fabricated by Laser Conversion and Cutting**

*Renxiao Xu*^1,2^*, Anton Zverev*^2^*, Aaron Hung*^2^*, Caiwei Shen*^1,2^*, Lauren Irie*^2^*, Geoffrey Ding*^2^, *Michael Whitmeyer*^2^*, Liangjie Ren*^2^*, Brandon Griffin*^2^*, Jack Melcher*^2^*, Lily Zheng*^2^*, Xining Zang*^1,2^*, Mohan Sanghadasa*^3^*, and Liwei Lin*^1,2^

^1^Department of Mechanical Engineering, University of California, Berkeley, CA 94720, USA

^2^Berkeley Sensor and Actuator Center, Berkeley, CA 94704, USA

^3^Aviation and Missile Research, Development, and Engineering Center, US Army, Redstone Arsenal, AL 35898, USA

Correspondence: Liwei Lin (lwlin@me.berkeley.edu)

# Supplementary Information

**Figure S1.** Top view optical (top) and 3D reconstruction images (bottom) of polyimide film surface after being irradiated by laser with different power levels. The scanning speed of laser head is fixed at 60 mm/s. Scale bars: 100 μm. The color in 3D reconstruction images denotes relative height above the top surface of the polyimide film.

**Figure S2.** (a) Sheet resistances and (b-f) SEM images (top: top view; bottom: cross-sectional view) of graphitic network structures obtained at different laser power levels. The scanning speed is fixed at 60 mm/s. Scale bars: 25 μm.

**Figure S3.** (a) Schematic illustration of a typical cutting slot structure, featuring the total heat affected region (gray arrow) and the cutting slot (red arrow). (b) The widths of total heat affected region and cutting slot for different laser power levels. (c-f) Top view optical images of polyimide film surface after being irradiated by laser with different power levels. The scanning speed of laser head is fixed at 60 mm/s.

**Figure S4.** Buckling determinism analysis of a representative SMSP. (a) The first four buckling modes of a SMSP shown in top view (top row), side view (middle row) and 3D view (bottom row). The color denotes out-of-plane displacement. (b) Critical buckling strain corresponding to the first four modes. (c) Deformation energy as a function of elongation for the first four modes. Mode I (shown in red) has both the smallest critical buckling strain and the lowest deformation energy.

**Figure S5.** (a) Schematic illustration of the experimental setup for measuring resistance of test sample during its bending deformation. (b) The relative change in resistance of the test sample as a function of the relative change in length, for both upward (blue) and downward (red) bending. (c) The FEA-predicted (top left in each group) and experimentally observed (top right in each group) deformation of the test sample at 50% compression. The color in the top view FEA image of the test sample denotes computed maximum absolute principal strain.

**Figure S6.** Deformation of the SMSP with “alternating offset cuts” design at elongations beyond 282.5%, predicted by FEA. The magnified view shows the distribution of maximum absolute principal strain $\varepsilon_{max}$ in the graphitic material, at the site with the largest deformation. $\varepsilon_{max}$ reaches 5.5% when the overall elongation is 510%. The circles highlight local regions where $\varepsilon_{max}$ is greater than 5.5% (with grey or black colors).

**Figure S7.** Optical images of the SMSP at different stages of deformation in a rough stretching test.

**Figure S8.** Deformation at four stages of the SMSP with the “double spiral” design, predicted by FEA.

**Figure S9.** Deformation at four stages of the SMSP with the “zigzag serpentine” design, predicted by FEA.


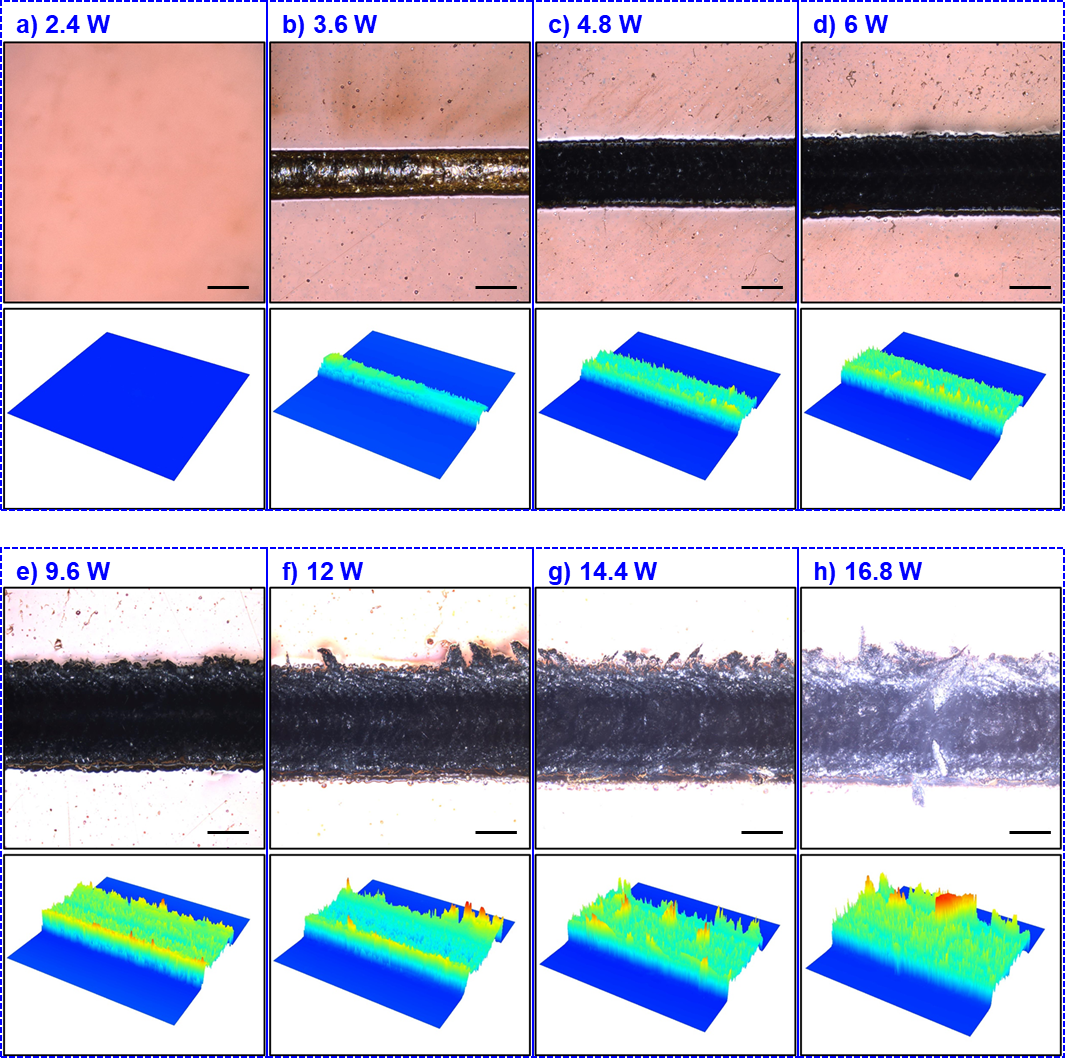


**Figure S1**


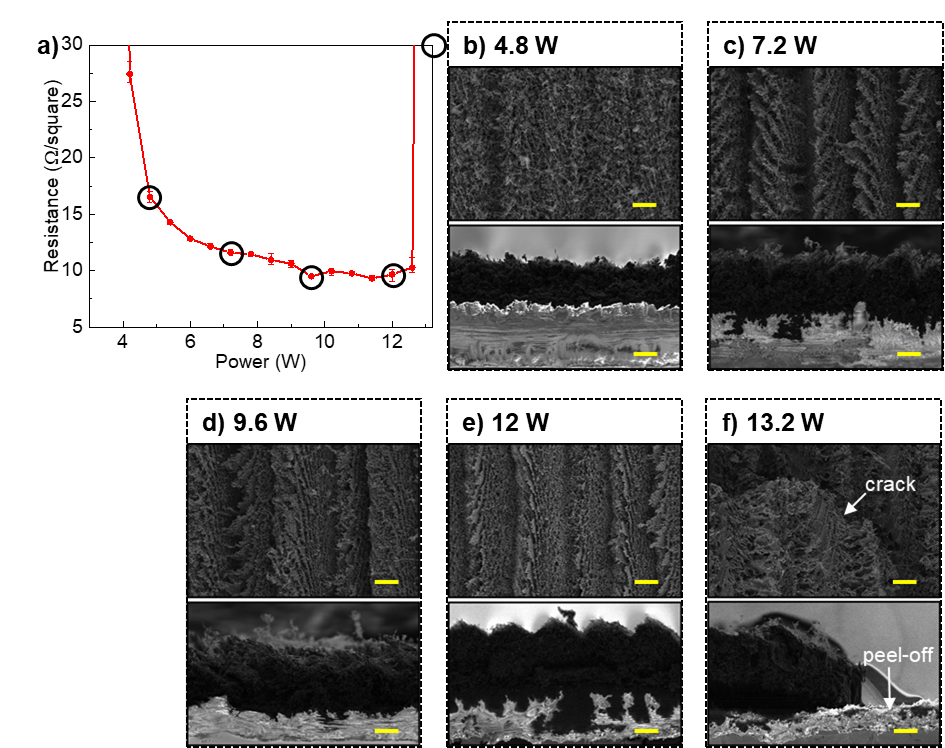


**Figure S2**


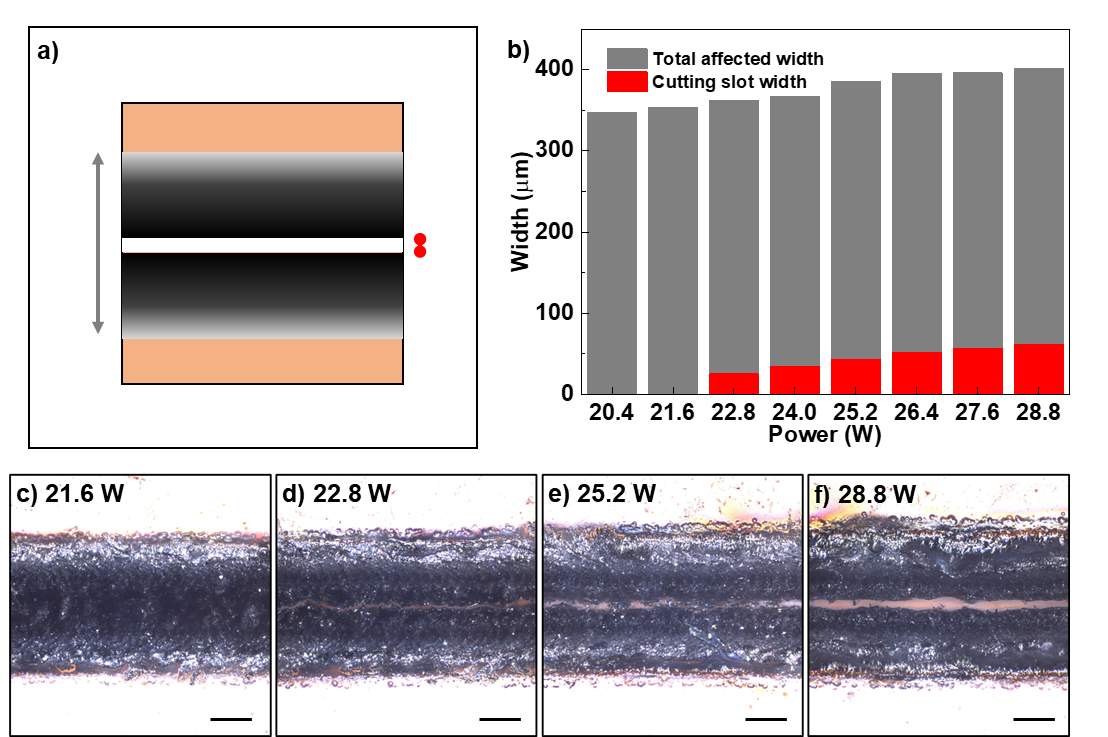


**Figure S3**


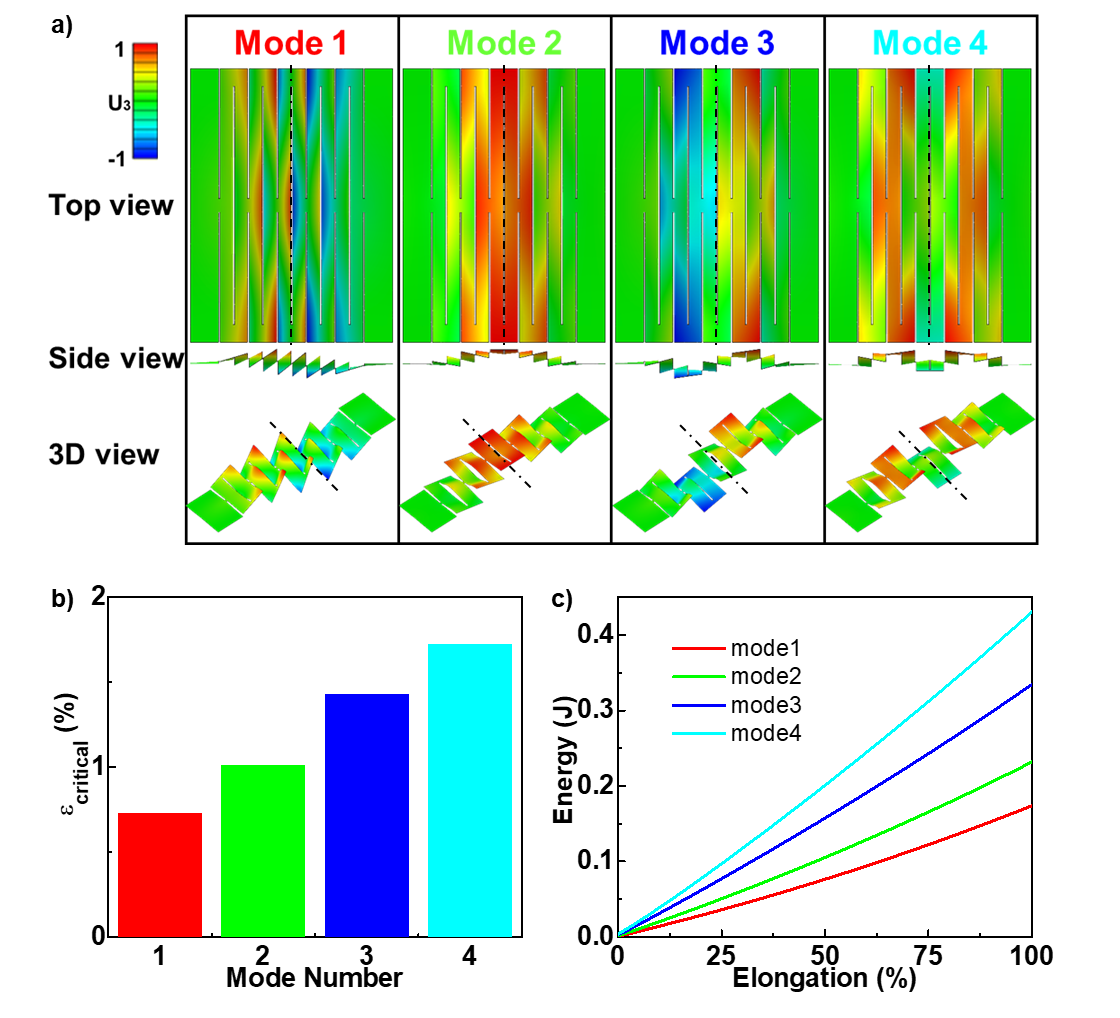


**Figure S4**


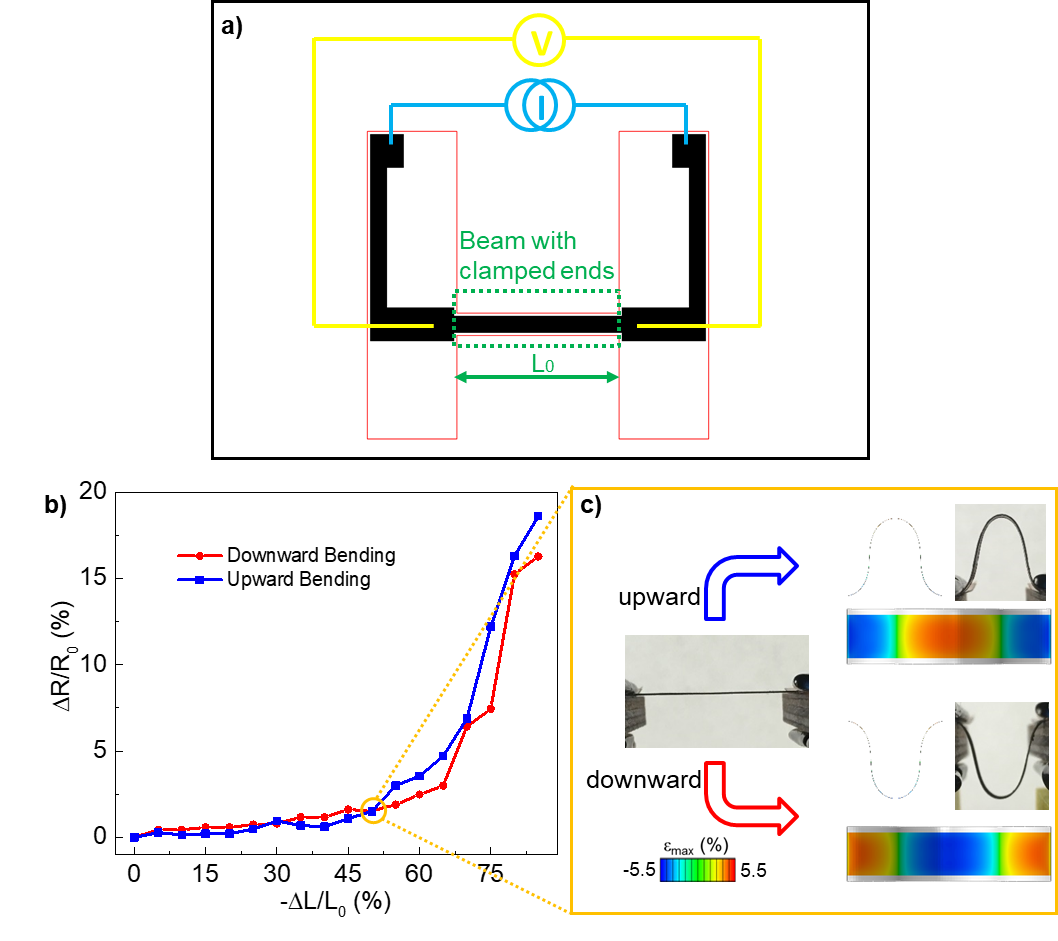


**Figure S5**


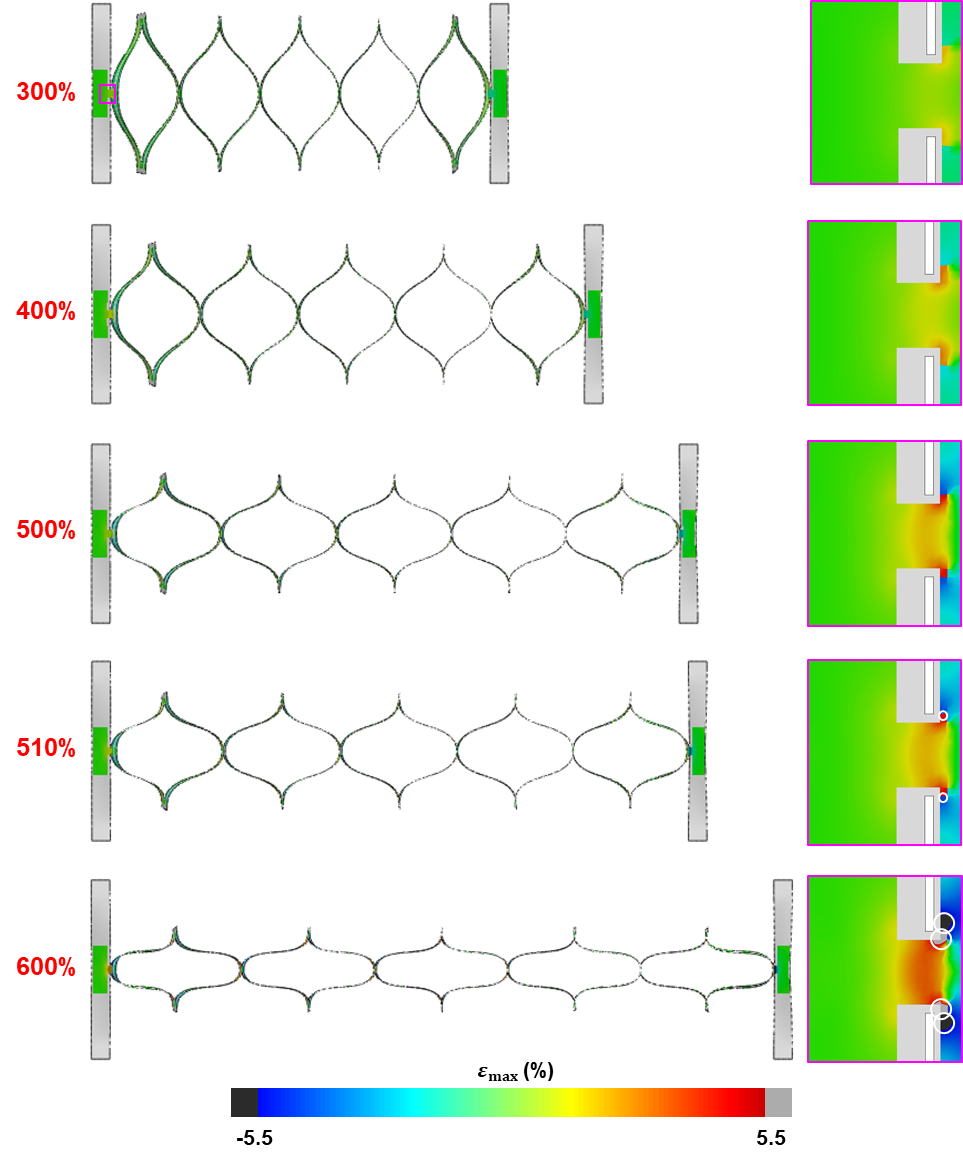


**Figure S6**


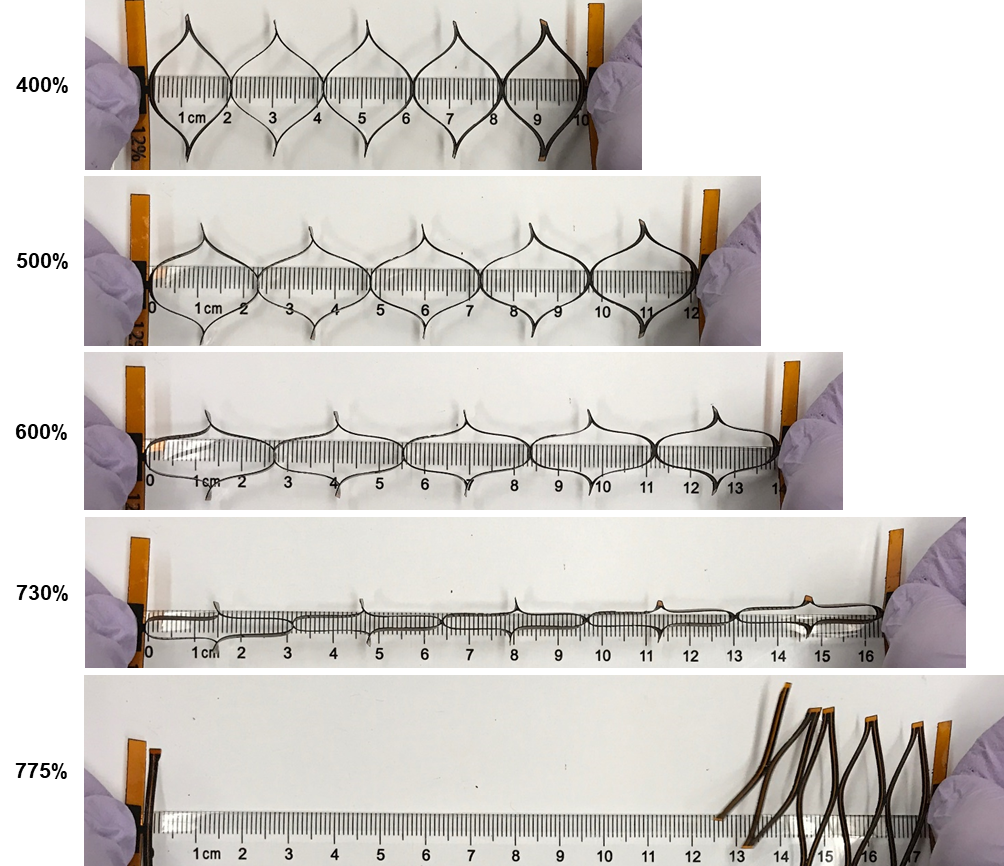


**Figure S7**


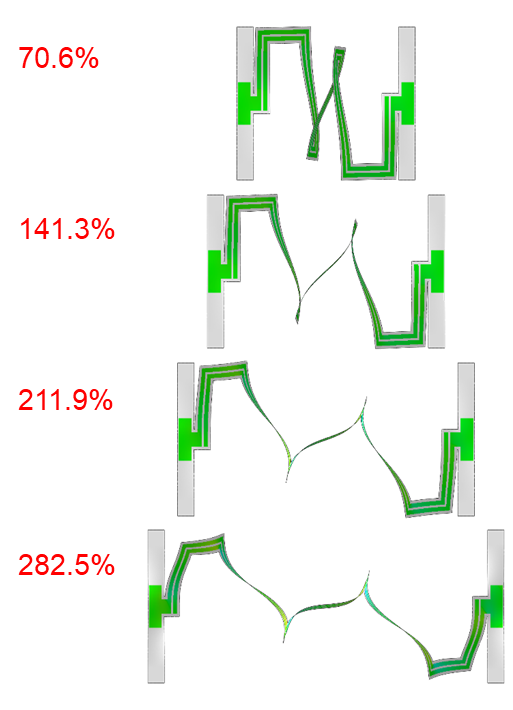


**Figure S8**


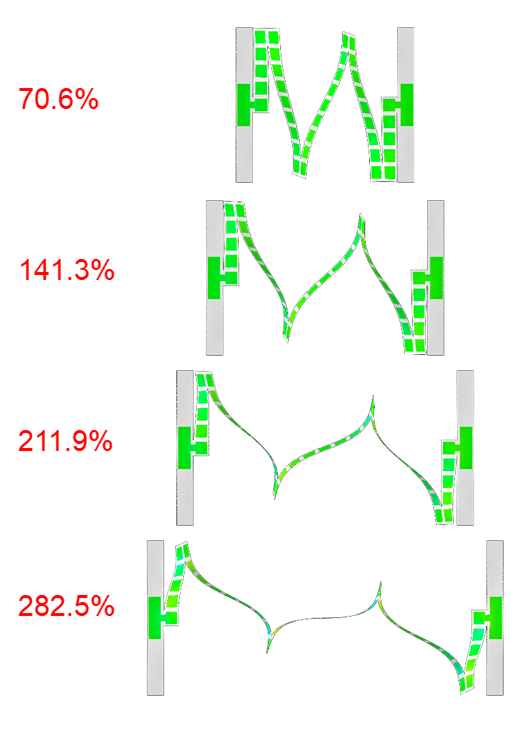


**Figure S9**
